# Profiling of Antimicrobial Metabolites Synthesized by the Endophytic and Genetically Amenable Biocontrol Strain Bacillus velezensis DMW1

**DOI:** 10.1128/spectrum.00038-23

**Published:** 2023-02-21

**Authors:** Chenjie Yu, Han Chen, Linli zhu, Yan Song, Qifan Jiang, Yaming Zhang, Qurban Ali, Qin Gu, Xuewen Gao, Rainer Borriss, Suomeng Dong, Huijun Wu

**Affiliations:** a Department of Plant Pathology, College of Plant Protection, Nanjing Agricultural University, Key Laboratory of Integrated Management of Crop Diseases and Pests, Ministry of Education, Nanjing, China; b Humboldt University Berlin, Institut für Biologie, Berlin, Germany; University of Minnesota Twin Cities

**Keywords:** *Bacillus velezensis* DMW1, whole-genome sequencing, secondary metabolites, plant growth promotion, disease control

## Abstract

The genus *Bacillus* is one of the most important genera for the biological control of plant diseases that are caused by various phytopathogens. The endophytic *Bacillus* strain DMW1 was isolated from the inner tissues of potato tubers and exhibited strong biocontrol activity. Based on its whole-genome sequence, DMW1 belongs to the Bacillus velezensis species, and it is similar to the model strain *B. velezensis* FZB42. 12 secondary metabolite biosynthetic gene clusters (BGCs), including two unknown function BGCs, were detected in the DMW1 genome. The strain was shown to be genetically amenable, and seven secondary metabolites acting antagonistically against plant pathogens were identified by a combined genetic and chemical approach. Strain DMW1 did significantly improve the growth of tomato and soybean seedlings, and it was able to control the Phytophthora sojae and Ralstonia solanacearum that were present in the plant seedlings. Due to these properties, the endophytic strain DMW1 appears to be a promising candidate for comparative investigations performed together with the Gram-positive model rhizobacterium FZB42, which is only able to colonize the rhizoplane.

**IMPORTANCE** Phytopathogens are responsible for the wide spread of plant diseases as well as for great losses of crop yields. At present, the strategies used to control plant disease, including the development of resistant cultivars and chemical control, may become ineffective due to the adaptive evolution of pathogens. Therefore, the use of beneficial microorganisms to deal with plant diseases attracts great attention. In the present study, a new strain DMW1, belonging to the species *B. velezensis*, was discovered with outstanding biocontrol properties. It showed plant growth promotion and disease control abilities that are comparable with those of *B. velezensis* FZB42 under greenhouse conditions. According to a genomic analysis and a bioactive metabolites analysis, genes that are responsible for promoting plant growth were detected, and metabolites with different antagonistic activities were identified. Our data provide a basis for DMW1 to be further developed and applied as a biopesticide, which is similar to the closely related model strain FZB42.

## INTRODUCTION

Plant diseases caused by plant pathogens, such as fungi, oomycetes, bacteria, nematodes, and viruses, are a major and long-standing threat to crop production. The agricultural production losses due to various plant diseases are estimated to be around 20 to 40%, every year, worldwide (1). To control plant diseases and ensure food production and quality, synthetic agrochemicals have been increasingly applied to agricultural crops over the past few decades. However, gains obtained through chemical input carry high economic and environmental costs, such as harmful environmental residues and the development of resistance in phytopathogens (2). The development and application of biological control agents (BCAs) is considered to be an environmental-friendly alternative to chemical pesticides (3). Plant growth-promoting rhizobacteria (PGPR) that have the ability to improve plant growth traits are a main source of BCAs. Among them, a specialized group of bacteria have evolved the ability to invade and live inside their host plant, thereby forming a subclass of PGPR, endophytic bacteria (4). These endosymbionts are able to promote plant growth directly and to protect the host from plant pathogens through indirect mechanisms, in a similar manner to rhizobacteria (5). Endophytic bacteria are beneficial to rhizobacteria because they live in a stable environment. This means that there are economic and ecological reasons to select endophytic bacteria for the development of BCAs (6).

The genus *Bacillus* is reported to be one of the most abundant genera in endophytic bacteria (7). It is a well-studied biocontrol microorganism, and it is considered to be the most promising and suitable candidate for the development of biopesticides in sustainable agriculture (8). *Bacillus* spp. can promote plant growth mainly via the enhancement of nutrient utilization and regulation of phytohormones. On the other hand, they have a strong ability to suppress and prevent plant diseases via the biosynthesis of antimicrobial substances as well as by stimulating induced systemic resistance (ISR) (9). Additionally, a few studies have demonstrated that some *Bacillus* strains can regulate the expression of plant stress-related genes to enhance the tolerance of their host plants against environmental stress, such as drought, salt, and heavy metal (10, 11). Members of the genus *Bacillus* utilize larger multi-enzyme complexes, such as non-ribosomal peptide synthetase (NRPS) and polyketide synthetase (PKS), to produce lipopeptides and polyketides with antimicrobial properties, respectively. The cyclic lipopeptides share similar structures and can be divided into three families on the basis of the amino acid sequence: iturins and fengycins with remarkable anti-fungal activity as well as surfactins that possess antibacterial, antiviral, and antimycoplasmal properties (12). Three PKS from *Bacillus* were identified as being involved in the biosynthesis of bacillaene, difficidin, and macrolactin, which were reported to have the capacity to suppress the growth of numerous pathogenic bacteria (13). Furthermore, bacilysin, which is a dipeptide antibiotic that is synthesized by NRPS, showed a high antagonistic effect against phytopathogens, such as Xanthomonas oryzae pv. *oryzae*, *X. oryzae* pv. *oryzicola*, and *P. sojae* (14, 15).

The aims of the present study are to identify and characterize the biocontrol endophytic bacterium DMW1 and to explore the mechanisms underlying its effect on plant growth promotion and disease control. Through a comparative genomic analysis, the identification of active metabolites, and greenhouse experiments, we designated the DMW1 strain as a member of *B. velezensis* and highlighted its potential as a biopesticide against Ralstonia solanacearum and *P. sojae.*

## RESULTS

### Biocontrol activity and phenotypic characterization.

The strain DMW1 was isolated from the inner tissues of surface-sterilized potato tubers. An *in vitro* antagonistic activity assay demonstrated a broad spectrum of antagonistic activity against various fungi (Botrytis cinerea, Sclerotinia sclerotiorum, Rhizoctonia solani, Fusarium graminearum, Fusarium verticillioides, Gaeumannomyces graminis, Pyricularia oryzae), oomycetes (*P. sojae*, *P. capsica*), and bacteria (*X. oryzae* pv. *oryzae*, Pantoea ananatis, R. solanacearum) (Fig. S1; Table S1). The isolate DMW1 produced various extracellular enzymes (protease, cellulase, and amylase), siderophore, and indole-3-acetic acid (IAA). These features of DMW1 were similar to those of the model biocontrol agent *B. velezensis* FZB42, which was isolated from the plant rhizosphere (Fig. S2) (16). Therefore, to gain a deeper knowledge of this strain, whole-genome sequencing of DMW1 was conducted.

### Sequencing and genome characteristics of *B. velezensis* DMW1.

**FIG 1:**
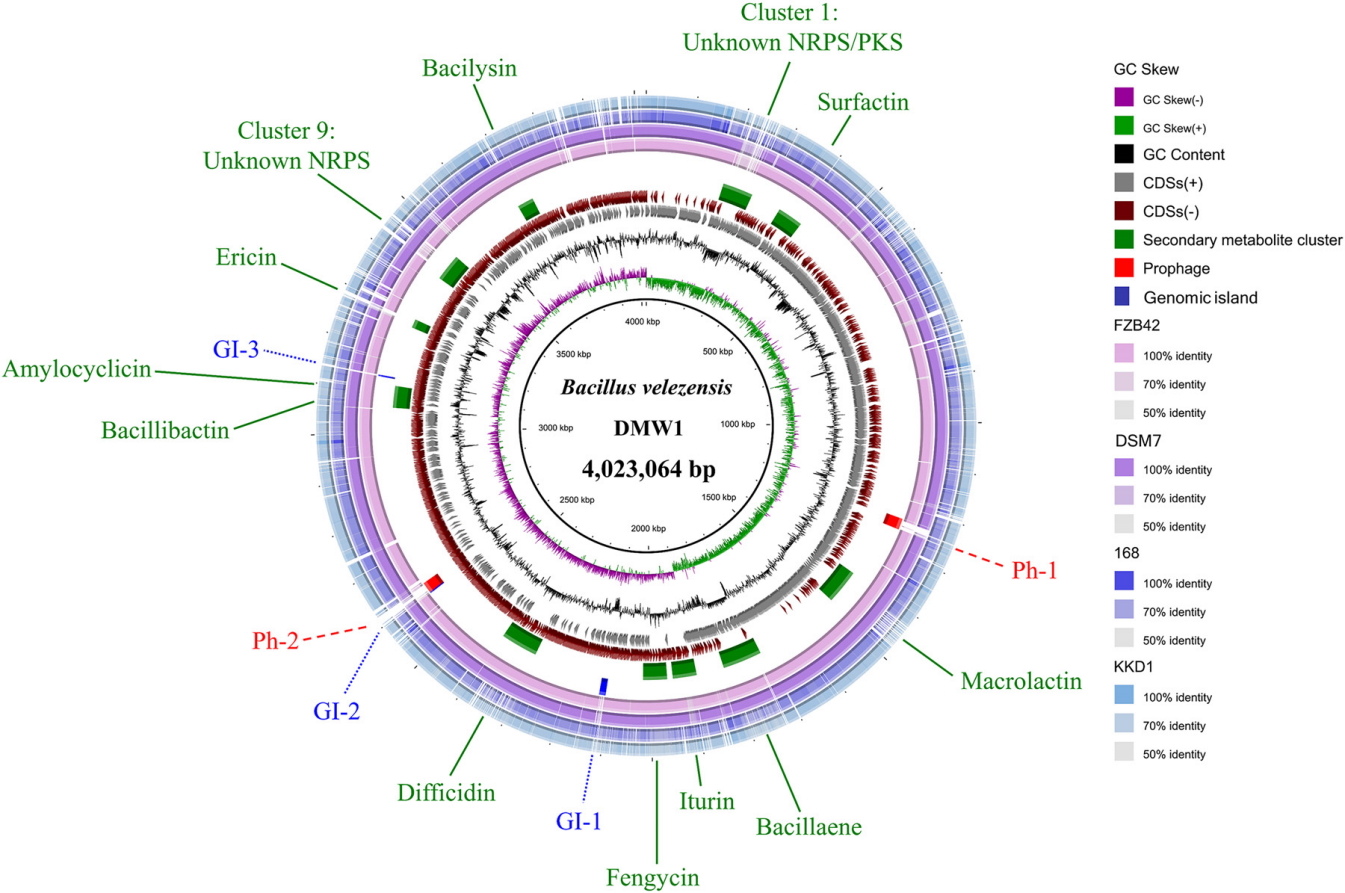
The complete genome map of *B. velezensis* DMW1 with its genomic features. The map consists of 10 circles. From the inner circle to the outer circle: (1) GC skew, (2) GC content, (3) genes transcribed in forward direction, (4) genes transcribed in reverse direction, (5) predicted secondary metabolite biosynthetic gene clusters, (6) predicted prophages and genomic islands, and (7 to 10) whole-genome BLAST of DMW1 with *B. velezensis* FZB42, *B. amyloliquefaciens* DSM7, B. subtilis 168, and *B. halotolerans* KKD1, respectively.

The genome sequencing of strain DMW1, using the Illumina Hiseq and PacBio platforms, yielded 123,684 total PacBio reads (1,589,853,672 bases) and 9,609,057 Illumina clean reads (1,448,266,351 bases). After a quality assessment by k-monomeric unit (k-mer) and a GC-depth analysis (Fig. S3 and S4), the complete genome was *de novo* assembled via Unicycler. DMW1 has a chromosome of 4,023,064 bp with 46.4% GC content, which is slightly larger than those of *B. velezensis* FZB42 and *B. amyloliquefaciens* DSM7 but is smaller than that of B. subtilis 168 and *B. halotolerans* KKD1 (Table 1). The genome of DMW1 was predicted to contain 4,236 genes, including 87 tRNA genes, 27 rRNA genes, 86 sRNAs, and 4,036 protein coding genes (CDSs). In total, 3,003 and 2,207 CDSs were annotated and assigned to 20 COG types and 41 KEGG pathways, respectively (Fig. S5 and S6). Notably, CDSs related to metabolism were found to be the most abundant type in both COG (40.96%) and KEGG (78.52%) analyses. They were dedicated to the transport and metabolism of nucleotides, amino acids, carbohydrates, lipids, and coenzymes, the biosynthesis of secondary metabolites, glycan, and xenobiotics, and energy production. These biological processes play a vital role in allowing biocontrol microorganisms to compete with pathogens and colonize in complex environments. The circular genome of DMW1 and a BLAST alignment with four other *Bacillus* are shown in Fig. 1. The genome of DMW1 was found to be most similar to that of FZB42, among all tested strains (Fig. 1). Mobile genetic elements (MGEs), including prophages (Ph), genomic islands (GI) and insertion sequences (IE), are an indispensable feature of bacteria and often participate in the genome evolution process (17). A bioinformatics analysis showed that DMW1 possessed two Phs and three GI regions, which contained the majority of the unique genes (singletons) (Fig. 1). In bacteria, these regions are associated with lateral gene transfer events (18, 19). Additionally, nine clustered regularly interspaced short palindromic repeat (CRISPR)-Cas systems were detected in the DMW1 chromosome. As another kind of MGE, the CRISPR-Cas system provides bacteria with acquired immunity against viruses (20).

**TABLE 1:** Genomic features of the *B. velezensis* DMW1 and other *Bacillus* species

Species	*B. velezensis* DMW1	*B. velezensis* FZB42	*B. amyloliquefaciens* DSM7	B. subtilis 168	*B. halotolerans* KKD1
Genome size (bp)	4,023,064	3,918,596	3,980,199	4,215,606	4,248,134
Protein coding genes	4,036	3,676	3,860	4,237	4,141
GC content (%)	46.4	46.5	46.1	43.5	43.6
Number of rRNAs	27	29	30	30	30
Number of tRNAs	87	88	93	86	86

### Strain DMW1 is a representative of *B. velezensis*.

**FIG 2:**
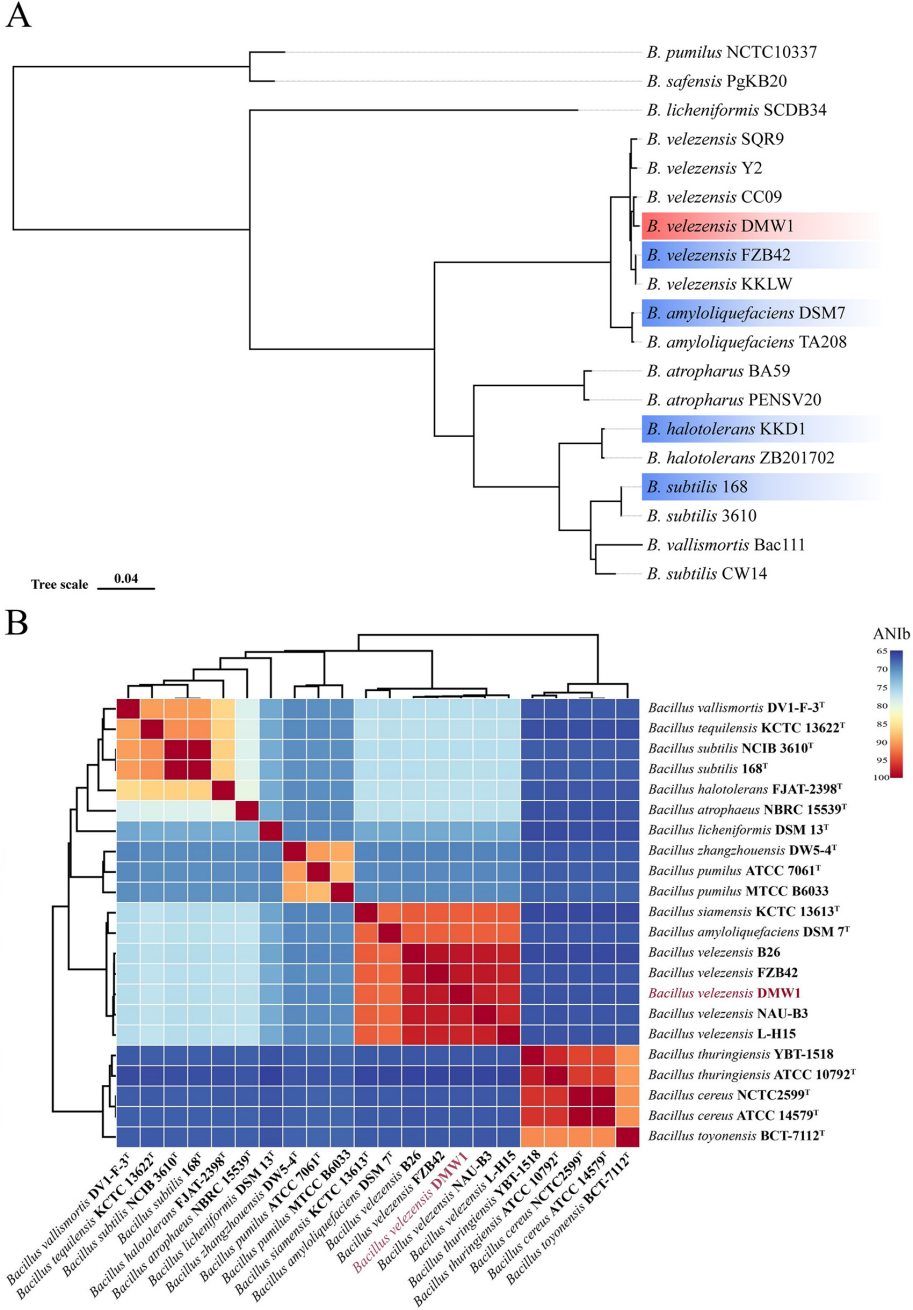
The phylogenetic analysis of *B. velezensis* DMW1. (A) The maximum likelihood phylogenetic tree was constructed using the 2,097 orthologous single-copy genes from 19 genomes of *Bacillus* species. The *B. safensis* PgKB20 and *B. pumilus* NCTC10337 were used as the outgroup. *B. velezensis* DMW1 are indicated in red, whereas the strains used for comparative analysis are indicated in blue. The numbers at the branch are bootstrapped values (%), based on 1,000 pseudo-replicates. (B) The heatmap based on the ANIb value of strain DMW1 and other *Bacillus* species. The *B. velezensis* DMW1 was labeled in red letters.

A phylogenetic tree based on the single-copy core genes from different *Bacillus* species revealed that the DMW1 strain is a member of the *B. velezensis* species (Fig. 2A). The most convincing method for prokaryotic species circumscriptions at the genomic level is the average nucleotide identity (ANI) between two genomes (21). In order to better understand the taxonomic status of DMW1, the approach of ANI based on BLAST (ANIb) was used, applying the online tool JSpeciesWS. According to the heat map in Fig. 2B, strain DMW1 was found to be assigned as a representative of *B. velezensis* species due to the high ANIb percentage (>96%). On the basis of these results, strain DMW1 was classified as a member of the *B. velezensis* species.

### Comparative genomic analysis of *B. velezensis* DMW1 with *Bacillus* strains.

**FIG 3:**
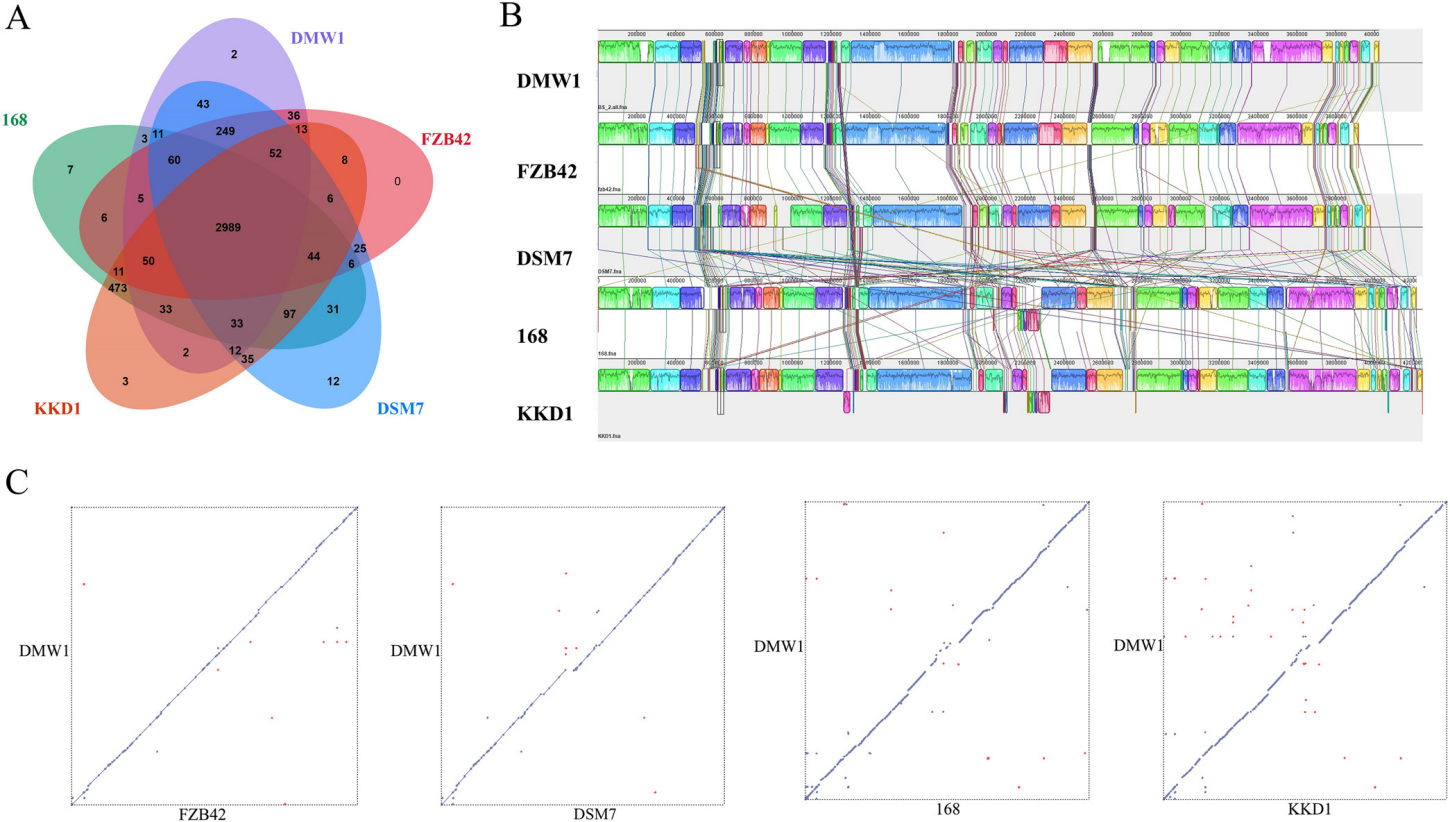
Comparative genomic analysis of *B. velezensis* DMW1 with other *Bacillus* strains (*B. velezensis* FZB42, *B. amyloliquefaciens* DSM7, B. subtilis 168, and *B. halotolerans* KKD1). (A) Venn diagram depicted the shared core orthologous gene families of all five strains as well as the unique gene families belonging to each strain. (B) Five-way whole-genome alignment of *Bacillus* strains using the Mauve software package. The locally collinear blocks (LCBs) with the same color represent conserved regions with synteny, and the lines connecting the LCBs indicate the translocation of conserved regions. The blocks above the horizontal lines indicate alignment in the forward direction, and those below the line indicate alignment in the negative direction. (C) Dot plots of the collinearity analysis between the chromosomes of DMW1 and FZB42/DSM7/168/KKD1 (from left to right). Each dot in the graph represents a maximal unique match (MUM). Purple dots indicate forward collinearity, whereas red dots indicate reverse collinearity.

The identification and comparison of orthologous genes among different strains is important for understanding the phylogeny and evolution of genes and genomes among different species (22). Therefore, a comparative analysis of the orthologous gene families was performed for the five *Bacillus* strains (Fig. 3A). The results revealed that the five genomes form 4,357 families, 1,389 orthologous families, and 2,968 single-copy gene families. Among them, 2,989 families (15,070 genes) were in the shared core orthologous gene families of all tested strains. A gene ontology enrichment analysis showed that the core gene families were associated with sporulation (GO:0030435) and with the plasma membrane (GO: 0005886). Notably, DMW1 and FZB42 shared 3,454 families with 95.68% identity, DMW1 and DSM7 shared 3,449 families with 92.02% identity, DMW1 and 168 shared 3,184 families with 74.6% identity, and DMW1 and KKD1 shared 3,184 families with 74.57% identity, indicating that a close homology between DMW1, FZB42, and DSM7 does exist.

The collinearity analysis of the whole-genome is an essential part of comparative genomics. Both multiple and pairwise alignments were conducted to further compare DMW1 with the other four *Bacillus* strains. The results demonstrated that the genomes of DMW1 and FZB42 shared the most locally collinear blocks (LCBs) with good synteny, suggesting an overall conserved genomic structure. On the contrary, numerous gene rearrangement and translocation events occurred between DMW1 and 168 or KKD1, pointing to their relatively far evolutionary distance from DMW1 (Fig. 3B). The pairwise collinearity analysis was visualized as a dot plot using MUMmer, and its results were consistent with those of the multiple alignments (Fig. 3C).

### Secondary metabolite biosynthetic gene clusters (BGCs) of *B. velezensis* DMW1.

**FIG 4:**
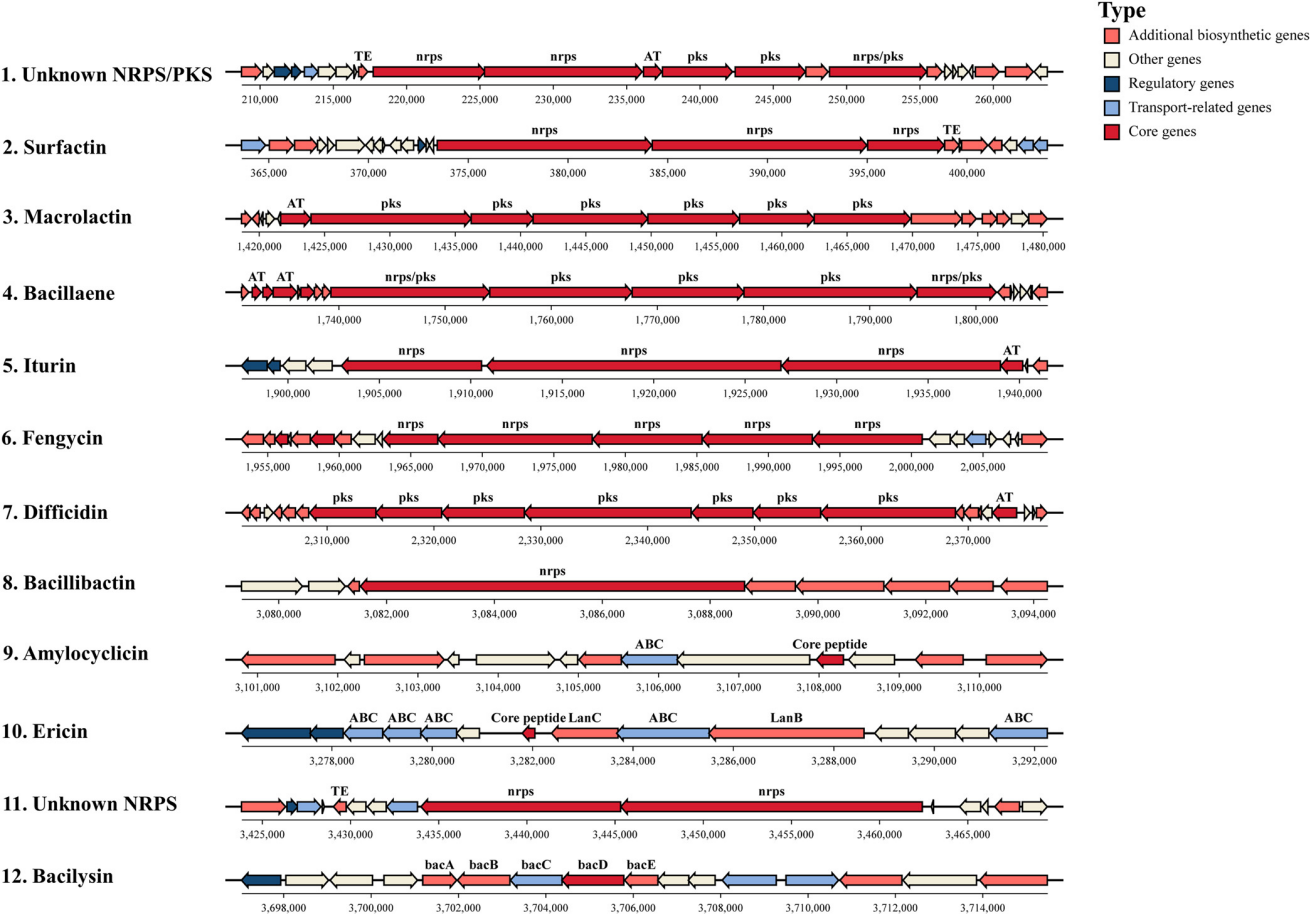
The presentation of secondary metabolite biosynthetic gene clusters in *B. velezensis* DMW1. In total, 12 BGCs were identified using the online tools antiSMASH and BAGEL4. Differently colored blocks indicate different types of genes. nrps, non-ribosomal peptide synthetase; pks, polyketide synthetase; AT, Acyltransferase; TE, Thioesterase; ABC, ABC transporter; lanB, dehydratase; lanC, cyclase; bacA–bacE, proteins responsible for the synthesis of bacilysin.

Secondary metabolites are the main biocontrol factor of *Bacillus* spp. that could directly suppress the phytopathogens that are present in the plant rhizosphere, and they are able to induce systemic resistance in host plants and thereby control the spread of plant diseases. By using antiSMASH and BAGEL4, a total of 12 BGCs were detected in the chromosome of DMW1, including 10 non-ribosomal and 2 ribosomal BGCs (Fig. 4). Among these gene clusters, five non-ribosomal peptide synthetases (NRPSs), three polyketide synthetases (PKSs), two ribosomal-synthesized and posttranslationally-modified peptides (Ripps) were identified as being dedicated to the biosynthesis of surfactin, iturin, fengycin, bacillibactin, bacilysin, macrolactin, bacillaene, difficidin, amylocyclicin, and ericin. DMW1 uses a large part of its genome (18.58%) for the biosynthesis, transport, and regulation of secondary metabolites. The comparison of BGCs in DMW1 with those in other *Bacillus* species is listed in Table 2. The distribution and arrangement of BGCs in the *B. velezensis* strains DMW1 and FZB42 were similar, due to the fact that 9 out of 12 clusters in DMW1 are well-conserved in the representatives of *B. velezensis* (23). The genomes of B. subtilis 168 and *B. halotolerans* KKD1 harbored a smaller set of conserved BGCs of DMW1. It is known that the members of the *B. amyloliquefaciens* operational group, especially *B. velezensis*, encompass a much greater number of secondary metabolites than do the other members of the B. subtilis species complex (24). This finding also corroborates a previous report that the distribution of the BGCs is closely related to the taxonomy of the genus *Bacillus* (25). Interestingly, we discovered three BGCs (clusters 1, 10, and 11) in DMW1 that were not present in any of the other four *Bacillus* strains that were used in the comparison. Among them, two clusters with unknown function (cluster 1 and cluster 11) were detected. Cluster 10 is involved in the biosynthesis, transport, and self-immunity of the lanthipeptide ericin, and it has been previously detected in *Bacillus* A1/3 and *B. velezensis* RC 218 (23, 26). Strain A1/3 has been originally described as member of the B. subtilis species, but it is now considered to be a member of the *B. velezensis* species (23).

**TABLE 2:** Secondary metabolic gene clusters in *B. velezensis* DMW1 and the comparisons with *B. velezensis* FZB42, *B. amyloliquefaciens* DSM7, B. subtilis 168, and *B. halotolerans* KKD1

*B. velezensis* DMW1				Presence (+) or absence (−) of gene clusters in other *Bacillus* strains			
Cluster number	Type	Size (Kb)	Most similar cluster (% of genes showing similarity)	FZB42	DSM7	168	KKD1
1	NRPS/PKS	77.61	Locillomycin (28%)	−	−	−	−
2	NRPS	65.09	Surfactin (86%)	+	+	+	+
3	PKS	87.82	Macrolactin (100%)	+	−	−	−
4	PKS	99.89	Bacillaene (100%)	+	+	+	+
5	NRPS	60.69	Iturin (88%)	+	+	−	−
6	NRPS	75.53	Fengycin (100%)	+	+	+	+
7	PKS	93.80	Difficidin (100%)	+	−	−	−
8	NRPS	40.53	Bacillibactin (100%)	+	+	+	+
9	Ripp	14.93	Amylocyclicin (100%)	+	−	−	−
10	Ripp	22.01	Ericin (100%)	−	−	−	−
11	NRPS	68.43	−^*a*^	−	−	−	−
12	NRPS	41.42	Bacilysin (100%)	+	+	+	+

a −, represents no similar cluster found via the antiSMASH analysis.

### Transformation efficiency of DMW1 is comparable with that of FZB42.

We transformed the GFP-plasmid (pAD 43-25) into strains DMW1, *B. velezensis* FZB42, and B. subtilis 168 to compare their transformation efficiencies. According to Fig. S7, DMW1 showed a comparable ability to take up plasmid DNA as FZB42, but its transformation efficiency was clearly less than that observed in B. subtilis 168.

### Isolation, identification, and bioactivity assay of secondary metabolites.

**FIG 5:**
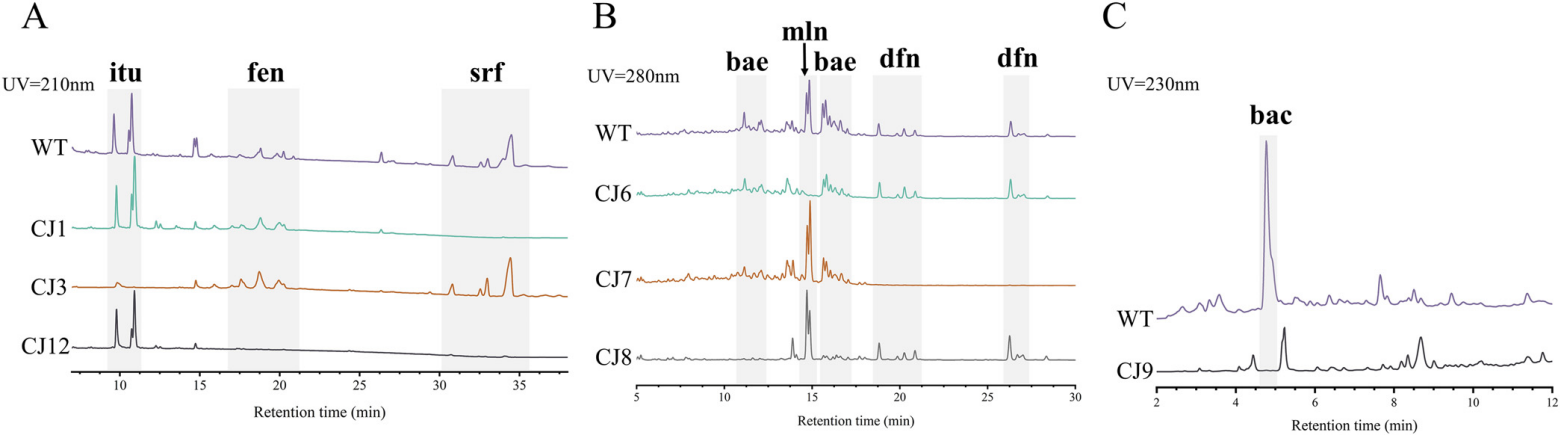
HPLC analysis of the metabolites in DMW1 and the mutant strains CJ1 (Δsrf), CJ3 (Δitu), CJ12 (Δsrf fen), CJ6 (Δmln), CJ7 (Δdfn), CJ8 (Δbae), and CJ9 (Δbac). WT, wild type DMW1; srf, surfactin; itu, iturin; fen, fengycin; mln, marcolactin; dfn, difficidin; bae, bacillaene; bac, bacilysin.

**FIG 6:**
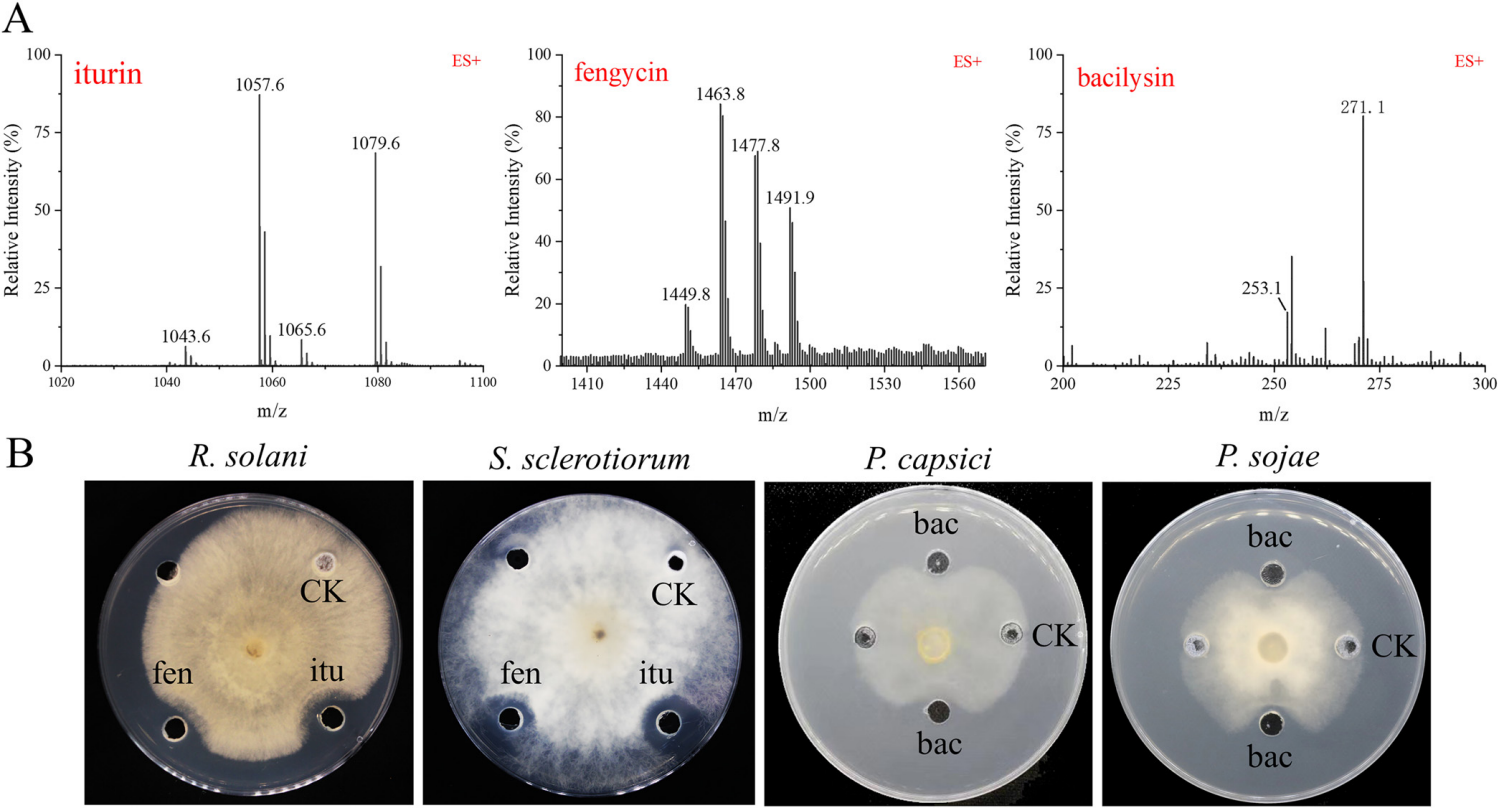
Identification of purified metabolites from DMW1 via UPLC-MS as well as their bioactivity assay. (A) Ions of *m/z* values 1043.6, 1057.6, and 1065.6 and 1079.6 correspond to C14–15 iturin A [M+H]+ and [M+Na]+, respectively. Ions of *m/z* values 1449.8, 1463.8, 1477.8, and 1491.8 correspond to C15–18 fengycin A [M+H]+. Ions of *m/z* values 271.1 correspond to bacilysin [M+H]+. (B) The antagonistic activity of iturin, fengycin, and bacilysin against phytopathogenic fungi and oomycetes. CK, methanol; itu, iturin; fen, fengycin; bac, bacilysin.

**FIG 7:**
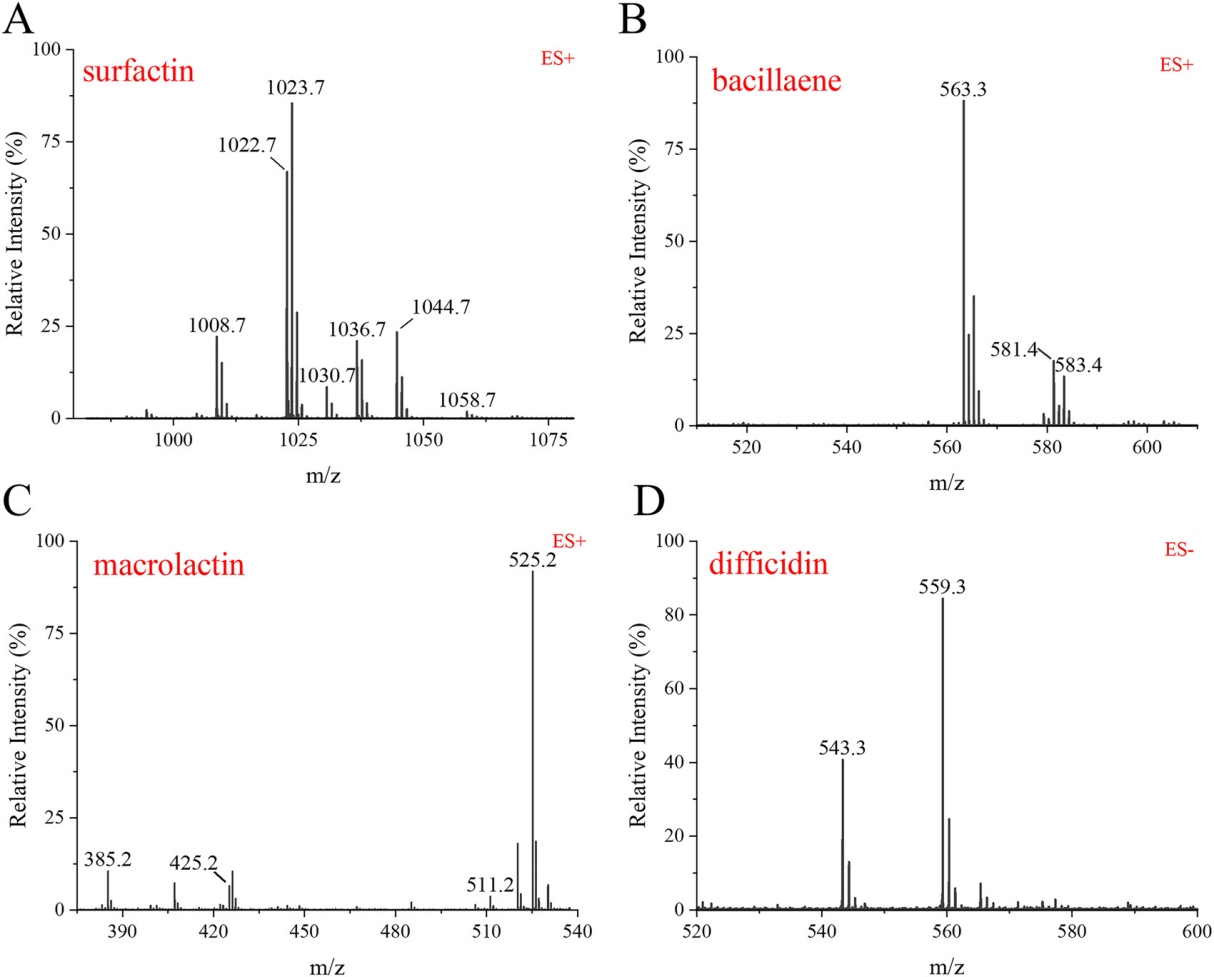
Identification of purified antibacterial metabolites from DMW1 via UPLC-MS. (A) Ions of *m/z* values 1008.7, 1022.7, and 1036.7 as well as 1030.7, 1044.7, and 1058.7 correspond to C13–15 surfactin A [M+H]+ and [M+Na]+, respectively. (B) Ions of *m/z* values 581.3 and 563.3 correspond to bacillaene [M+H]+ and [M-H_2_O+H]+. (C) Ions of *m/z* values 425.2 and 385.2 correspond to marcolactin A [M+Na]+ and [M-H_2_O+H]+. Ions of *m/z* values 511.2 and 525.2 correspond to 7-O-malonyl marcolactin A [M+Na]+ and 7-O-succinyl marcolactin A [M+Na]+, respectively. (D) Ions of *m/z* values 543.4 and 559.3 correspond to difficidin [M-H]+ and oxydifficidin [M-H]+, respectively.

The approach of genome mining combined with molecular manipulation was used to identify and isolate the likely antimicrobial compounds secreted by DMW1. According to the antiSMASH analysis of the BGCs (Fig. 4), seven identified BGCs that were involved in the biosynthesis of surfactin, iturin, fengycin, macrolactin, bacillaene, difficidin, and bacilysin were selected for knockout by using the markerless gene editing CRISPR-Cas9 system, as described in Materials and Methods. Via a high-performance liquid chromatography (HPLC) analysis of the wild-type and mutant strains, the peaks and retention times representing different secondary metabolites were ascertained (Fig. 5). These results indicated that DMW1 has the ability to produce the corresponding seven metabolites. All seven metabolites were then purified via resin adsorption and semi preparative reverse-phase HPLC. Via the use of an ultraperformance liquid chromatography-mass spectrometry system (UPLC-MS) and a comparison of the corresponding molecular ion peaks of each single metabolite to previously reported values, the seven metabolites were successfully purified (Fig. 6 and 7) and used for further activity detection.

To elucidate the role of lipopeptides and polyketides in the biocontrol of DMW1, the purified compounds were evaluated for antimicrobial activity against phytopathogens (Fig. 6B; Table 3). The results showed that only iturin and fengycin displayed antifungal activity. The inhibition rates of 50 μg/mL iturin and fengycin against R. solani were 54.63% and 50.5%, respectively. However, they did not exert any antagonistic activity against oomycetes or bacteria. Antibacterial activity was exerted by surfactin, the three polyketides (macrolactin, bacillaene, and difficidin), and bacilysin. Among these metabolites, difficidin showed the lowest MIC values against three bacteria, which were 4 μg/mL for *X. oryzae* pv. *oryzae* and 8 μg/mL for R. solanacearum and *P. ananatis*. As for oomycetes, only the dipeptide bacilysin displayed an inhibitory effect on *P. capsica* (53.75%) and *P. sojae* (44%), which means that bacilysin is the major substance in DMW1 against oomycetes. Taken together, DMW1 used different metabolites to combat different kinds of phytopathogens.

**TABLE 3:** Bioactivity assay of antimicrobial compounds produced by *B. velezensis* DMW1^*a*^

Phytoathogen	Inhibition rate (%) or MIC (μg/mL)						
	Itu	Fen	Srf	Mln	Dfn	Bae	Bac
Fungi							
R. solani	54.63%	50.5%	—	—	—	—	—
*S. sclerotiorum*	47.93%	43.62%	—	—	—	—	—
Oomycetes							
*P. capsici*	—	—	—	—	—	—	53.75%
*P. sojae*	—	—	—	—	—	—	44%
Bacteria							
*X. oryzae* pv. *oryzae*	>256	>256	32	128	4	64	16
R. solanacearum	>256	>256	32	64	8	64	32
*P. ananatis*	>256	>256	16	32	8	16	32

a Itu, iturin; fen, fengycin; srf surfactin; mln, macrolactin; dfn difficidin; bae, bacillaene; bac, bacilysin. The concentration of each metabolite used for the antifungal and antioomycetes assay was 50 μg/mL. —, no inhibition.

### Growth promotion effect of *B. velezensis* DMW1 on soybean and tomato plants.

Our whole-genome analysis discovered a series of genes associated with plant growth promotion in DMW1, including nutrient utilization, the synthesis of plant growth hormone, VOCs, and siderophores, suggesting the potential role of DMW1 in the promotion of plant growth (Table S2). Therefore, greenhouse experiments were conducted to test the effect of strain DMW1 on the growth traits of soybean and tomato seedlings (Table 4). The results showed a significant increase in the soybean stem height of 10.8%, root length of 24.14%, fresh weight of 20.3%, and dry weight of 28.57% in the seedlings that were treated with the DMW1 strain, compared to untreated seedlings. Moreover, the growth promotion effect by DMW1 on soybean stem height and root length is greater than observed with those that were treated with the model FZB42. In the tomato *in planta* experiment, the strain DMW1 increased the fresh and dry weight of seedlings by 34.33%, and 45.1%, respectively. Additionally, the height and thickness of the stem were more improved by DMW1 than by FZB42. In general, DMW1 was shown to have a similar ability to that of FZB42 to improve soybean and tomato growth traits.

**TABLE 4:** Effect of *B. velezensis* DMW1 and *B. velezensis* FZB42 on the growth traits of soybean and tomato seedlings^*a*^

Strain	Soybean				Tomato			
	Stem height	Root length	Plant fresh weight	Plant dry weight	Stem height	Stem thickness	Fresh weight (aerial parts)	Dry weight (aerial parts)
CK	31.5 ± 4.05^c^	12.8 ± 3.69^c^	1.97 ± 0.443^b^	0.21 ± 0.064^b^	31.65 ± 5.79^c^	0.41 ± 0.036^c^	5.71 ± 2.02^b^	2.04 ± 1.23^b^
DMW1	34.9 ± 4.01^a^	15.89 ± 4.59^a^	2.37 ± 0.474^a^	0.27 ± 0.093^a^	38.06 ± 4.61^a^	0.51 ± 0.040^a^	7.67 ± 1.67^a^	2.96 ± 1.52^a^
FZB42	33.31 ± 3.41^b^	13.63 ± 3.48^b^	2.26 ± 0.488^a^	0.25 ± 0.071^a^	35.3 ± 6.20^b^	0.46 ± 0.035^b^	7.27 ± 1.91^a^	2.77 ± 1.42^a^

a CK, the liquid LB medium was set as the control. The data were analyzed by a one-way ANOVA that was followed by Duncan’s multiple range test. Values represent the mean ± SEM of three independent experiments. Different lowercase letters within the rows represent statistically significant differences (α = 0.05).

### Biocontrol effect of *B. velezensis* DMW1 in soybean and tomato plants infested with phytopathogens.

**FIG 8:**
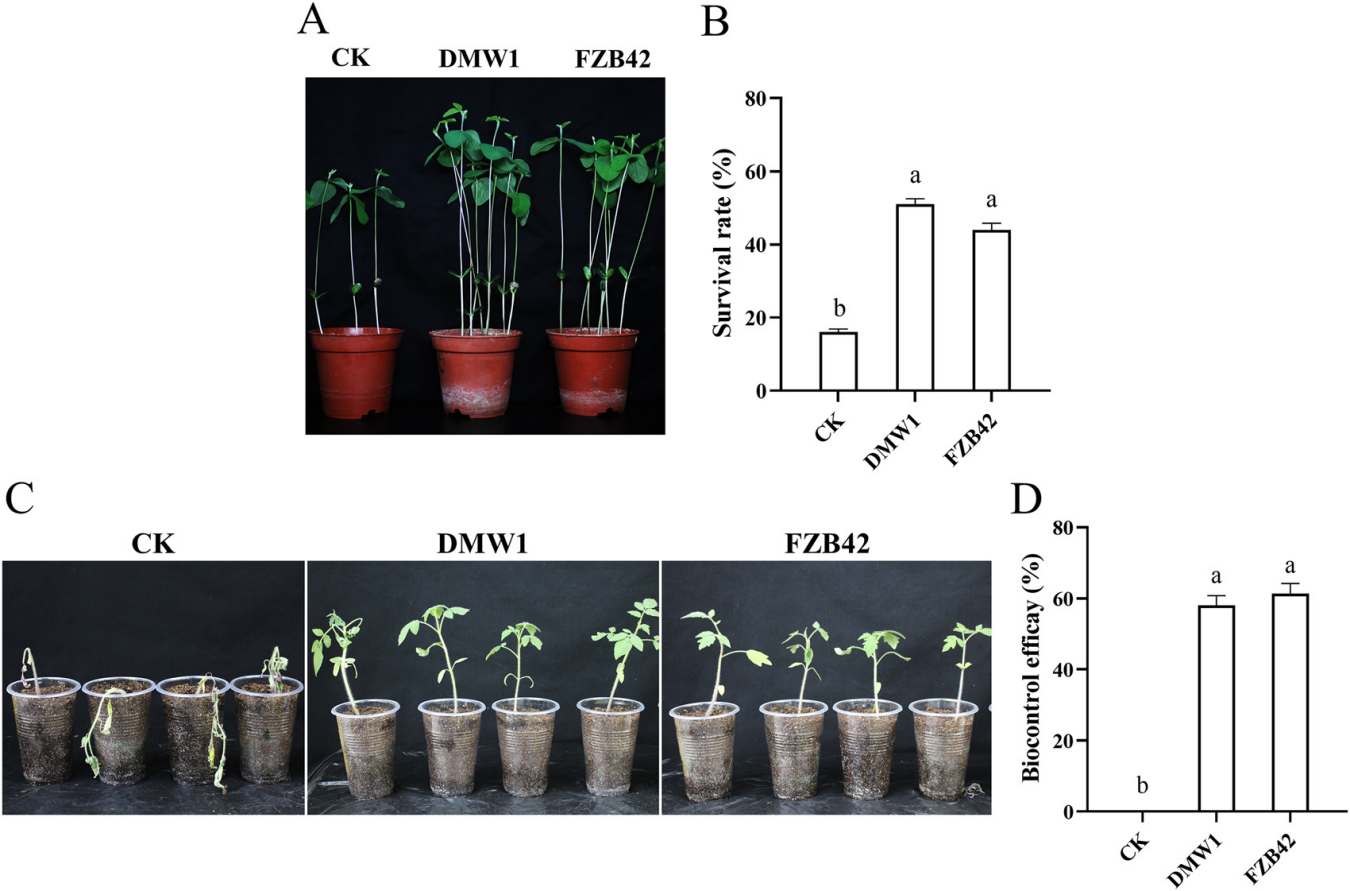
Biocontrol effect of *B. velezensis* DMW1 and *B. velezensis* FZB42 in plants. Photographs of soybean (A) and tomato (C) that were treated with DMW1 and FZB42 under *P. sojae* and R. solanacearum infection. CK, liquid LB medium was used as the control. The survival rates of soybean were calculated to determine the biocontrol efficacy of DMW1 and FZB42 against *P. sojae* (B). The biocontrol efficacy (D) was recorded 14 days after inoculation with R. solanacearum. The data represent the mean ± SEM of independent experiments. Different lowercase letters within the rows represent statistically significant differences (α = 0.05).

Greenhouse pot experiments were conducted to prove the biocontrol effect of DMW1 on the pathogenicity of *P. sojae* and R. solanacearum in soybean and tomato plants. Strain FZB42 was used as a reference. The results showed that the survival rates of soybean after being treated with DMW1 and FZB42 were 51% and 44%, which were significantly higher than those observed in the untreated control (16%), suggesting that the DMW1 and FZB42 strains exhibited similar control efficiency against *P. sojae* infections in soybean (Fig. 8A and B). Furthermore, under the infection of the soilborne plant pathogen R. solanacearum, the untreated tomato plants turned yellow, whereas the plants treated with DMW1 remained mostly green and healthy. The biocontrol efficacy of DMW1 was 58%, which is similar to the treatment efficacy of FZB42, indicating the strong capability of DMW1 to reduce the bacterial wilt disease in tomato in the same manner as FZB42 (Fig. 8C and D).

## DISCUSSION

*Bacillus* spp. are well-known bacteria that enhance plant growth and inhibit the growth of phytopathogens, thus making them a promising alternative to chemical pesticides. It has been reported that nearly half of the commercially available biocontrol bacterial products involve *Bacillus* strains. In the current study, we investigated the endophytic bacterium DMW1, isolated from the inner tissues of potato tubers, because of its striking capacity to inhibit the growth of various phytopathogens *in vitro*, including fungi, oomycetes, and bacteria (Fig. S1; Table S1). Its antagonistic activity, together with the production of macromolecule-degrading enzymes (protease, cellulase, and amylase), siderophores, and IAA (Fig. S2), as well its ability to take up DNA, recommended the strain DMW1 as a highly promising BCA and as a microbial factory in which to produce commercial enzymes.

In the present study, through the use of third-generation sequencing technology, the whole-genome sequence of the DMW1 strain was obtained in high quality. Taking advantage of the genome data of DMW1 and other *Bacillus* strains, a phylogenetic analysis was conducted. The strain DMW1 was classified as a member of the *B. velezensis* species, according to the phylogenetic tree based on the core genome and the heat map of the ANIb values (Fig. 2). *B. velezensis* was first isolated and described by Ruiz-Garcia et al. (27), and it was designated to be a member of the operational group *B. amyloliquefaciens* (24). A comparative genomic analysis revealed that DMW1 was most similar to *B. velezensis* FZB42 in terms of its genomic structure and gene function, which explains why DMW1 and FZB42 have similar biocontrol characteristics (Fig. 3). DMW1 possesses 4,036 coding genes, which is more than are possessed by FZB42 (3,676), which is partly due to the prophage insertion that is present in the DMW1 genome.

*B. velezensis* strains have been increasingly studied and applied due to their strong ability to produce bioactive secondary metabolites (28). Nine well conserved BGCs, which direct the synthesis of lipopeptides (surfactin, iturin, and fengycin), polyketides (macrolactin, bacillaene, and difficidin), siderophores (bacillibactin), dipeptides (bacilysin) and peptides (amylocyclicin), are present in the members of *B. velezensis* (23). 12 BGCs were predicted in strain DMW1, and the related gene size was up to 0.74 MB (Fig. 4). In addition to nine conserved BGCs, strain DMW1 possess two gene clusters that are not present in the other four *Bacillus* strains, and they may be responsible for the production of novel compounds.

Non-ribosomal peptide and polyketide are two main types of antimicrobial substances produced by *Bacillus* spp. (13). To clarify their roles in the antimicrobial activity of DMW1 against various phytopathogens, seven antimicrobial substances were isolated, and their bioactivity spectra were detected (Fig. 6 and 7; Table 3). The antifungal assay showed that two lipopeptides, namely, iturin and fengycin, can inhibit the growth of *S. sclerotiorum* and R. solani, which is consistent with previously reported results (29, 30). The dipeptide bacilysin is the only compound that was found to be responsible for the inhibitory action against the oomycetes *P. capsica* and *P. sojae*, corroborating a recent study that found that FZB42 could produce bacilysin to suppress the growth of *P. sojae* by affecting the expression of genes related to growth, macromolecule biosynthesis, pathogenicity, and ribosomes (15). The polyketides produced by *Bacillus* are considered to be inhibitors of a variety of multidrug-resistant and phytopathogenic bacteria, including methicillin-resistant Staphylococcus aureus (31), R. solanacearum (32, 33), and Erwinia amylovora (34). According to our results, all three polyketides (macrolactin, bacillaene, and difficidin) displayed bactericidal activity against *X. oryzae* pv. *oryzae*, R. solanacearum, and the causative agent of maize brown rot, namely, *P. ananatis* (35), corroborating earlier findings that were summarized by Chowdhury et al. (36). Moreover, the minimum inhibitory concentration (MIC) values of surfactin and bacilysin against these phytopathogenic bacteria were around 16 to 32 μg/mL, suggesting their important role in the antibacterial action of DMW1.

Many members of *Bacillus* species have been reported to be plant growth promoters. FZB42 has been characterized as promoting plant growth via the secretion of indole-3-acetic acid (37). Batista et al. applied *Bacillus* sp. RZ2MS9 in corn and soybean seedlings under greenhouse conditions to achieve more than a 47% increase of shoot dry weight (38). *B. amyloliquefaciens* BS006 was found to promote the growth of banana, with the growth parameters being comparable to those of 100% fertilization (39). In general, the microbial treatment with DMW1 could significantly facilitate the growth of soybean and tomato seedlings in greenhouses, leading to an increase in stem height, root length, and plant weight (Table 4). This is in agreement with the fact that DMW1 possesses many growth promotion related genes that are involved in nitrogen fixation, spermidine synthesis, and auxin production (Table S2). *P. sojae* and R. solanacearum, the causative agent of soybean root rot and tomato bacterial wilt, are two well-known phytopathogens that cause significant decreases in crop yields and economic losses worldwide. Due to the favorable *in vitro* antagonistic activity of DMW1 against these two phytopathogenic microorganisms, *in planta* experiments were performed. The results indicated that the infection of *P. sojae* and R. solanacearum on soybean and tomato are well-controlled by the application of DMW1 (Fig. 8). Taken together, the biocontrol efficiency of DMW1 is comparable with that of FZB42, suggesting DMW1 as a promising resource for the biocontrol of diseases caused by *P. sojae* and R. solanacearum.

In summary, we have reported that the potato endophyte *B. velezensis* DMW1 possessed outstanding biocontrol features, such as plant growth promotion in soybean and tomato, and did suppress various plant pathogens. Seven different secondary metabolites with antagonistic activity were identified via a combined approach of site directed mutagenesis, applying the CRISPR-Cas9 methodology, and chemical profiling by mass spectrometry. The compounds isolated from DMW1 were found to act antagonistically against bacterial and fungal plant-pathogens, including oomycetes. Due to its accessibility to genetic manipulation, DMW1 is a promising candidate for the study of the effects of endophytic biocontrol bacteria on plant health, and it could be considered to be a counterpart for comparative investigations with the rhizobacterium FZB42, which is only able to colonize root surfaces and is not able to penetrate into inner plant tissues (40, 41).

## MATERIALS AND METHODS

### Microbial strains and growth conditions.

The strains and plasmids used in this work are listed in Table S3, and all of the primers that were used are listed in Table S4. The DMW1 strain was isolated from potato tubers that were collected from Guizhou, China. The potato tubers were cleaned in running water and sterilized with 70% ethanol and 2% sodium hypochlorite for 1 min. Subsequently, the surface sterilized samples (10 g) were crushed using an automatic sample grinder (Tissuelyser-64, Jinxin Ltd., Shanghai, China) and suspended in 50 mL sterile distilled water. The mixed solution was incubated for 30 min at 37°C under gentle shaking and was then heated for 10 min at 80°C. The samples were serially diluted and plated onto LB plates at 37°C for 15 h. After incubation, the individual colonies were subcultured on LB plates to obtain pure colonies. The strains were stored in glycerol stocks at –80°C until future use.

All of the fungi were routinely cultivated in PDA medium at 25°C, whereas the oomycetes were grown in 10% (vol/vol) V8 juice medium. Bacillus velezensis DMW1 underwent standard growth in Luria Bertani (LB) medium at 37°C, and the other bacterial strains were cultured in NB medium at 30°C.

### Whole-genome sequencing, assembly, and analysis.

The genomic DNA of *B. velezensis* DMW1 was isolated and purified using the FastPure Bacteria DNA Isolation Kit (DC112, Vazyme Biotech Co., Ltd.), following the manufacturer’s instructions. The whole-genome sequencing was performed on Illumina Hiseq 4000 and PacBio SMRT platforms (Shanghai Majorbio Bio-pharm Technology Co., Ltd.). A k-mer analysis was carried out using Meryl software (https://github.com/marbl/meryl/) to evaluate the size of the genome. Meanwhile, a GC-depth analysis was carried out by Bowtie2 (version 2.4.5, http://bowtie-bio.sourceforge.net/bowtie2/index.shtml) to obtain the GC distribution. The software Unicycler (version 0.4.8) was used to *de novo* assemble the complete genome. The genes of DMW1 were predicted using Glimmer (version 3.02, http://ccb.jhu.edu/software/glimmer/index.shtml). The tRNA (tRNA) genes were predicted via tRNAscan-SE (version 2.0, http://trna.ucsc.edu/software/). The ribosome RNA (rRNA) genes were predicted via Barrnap (version 0.8, https://github.com/tseemann/barrnap/). The small RNA (sRNA) was predicted via Infernal software (version 1.1.4, http://eddylab.org/infernal) and the Rfam database (https://rfam.org/). The annotation and classification of all CDSs were performed using the EggNOG database (http://eggnog.embl.de/) and the Kyoto Encyclopedia of Genes and Genomes database (KEGG, http://www.genome.jp/kegg/). The prophage, genomic island and CRISPR-Cas system was detected using Islander (version 1.2, http://www.pathogenomics.sfu.ca/islandviewer/). The circular map consists of the DMW1 genome, and a BLAST comparison was generated using the BLAST Ring Image Generator software package (BRIG, version 0.95) (42). The secondary metabolite biosynthetic gene clusters (BGCs) were predicted by antiSMASH (43) and BAGEL4 (44).

### Phylogenetic analysis of *B. velezensis* DMW1.

The orthologous single-copy genes of the genomes were calculated using the OrthoFinder (45), followed by the multiple alignments of the above genes using MAFFT (46). After that, Python (version 3.7) was applied to concatenate all alignments into one large alignment. Finally, the concatenated alignment was calculated by IQ-TREE using the LG+I+G model to generate a maximum likelihood phylogenetic tree in Newick format (47). The average nucleotide identity based on BLAST (ANIb) was calculated by pairwise genome comparisons using JSpeciesWS (48), and the genome data were acquired from the National Center for Biotechnology Information (NCBI, http://www.ncbi.nlm.nih.gov/).

### Comparative genomic analysis.

An orthologous gene family analysis of the whole-genome was performed using the online tool OrthoVenn2 with the following parameters: E value, 1E−2; inflation value, 1.5 (https://orthovenn2.bioinfotoolkits.net/home) (49). A genome-to-genome alignment was conducted using the Mauve software package (version 2.4.0) with the default parameters (50). *B. velezensis* DMW1 was set as the reference genome. MUMmer (version 3.23), running on Linux, was used to perform pairwise alignments of the entire genome (51).

### Construction of the *B. velezensis* DMW1 mutants.

For the construction of the DMW1 mutants, a 1,000 bp sequence deletion of a core gene from the BGCs was introduced into the genome using the markerless CRISPR-Cas9 system described by Altenbuchner (52), with some modifications. We used the original plasmid pJOE8999 to generate knockout vectors. First, single-guide RNA (sgRNA) was predicted using guide design resources (https://zlab.bio/guide-design-resources) and integrated into the BsaI restriction sites of pJOE8999. In a second step, upstream and downstream fragments of targeted regions with lengths of 800 bp were amplified, spliced, and inserted into the SfiI sites to generate the knockout vectors. The DNA transformation of the DMW1 strain was then conducted, according to a previously described method (53). Single colonies appearing on LB plates containing 50 μg/mL kanamycin and 0.2% mannose (for the activation of the cas9 gene) were detected via colony PCR and sequencing. To eliminate the vector, the positive colonies were then cultured in LB liquid medium without antibiotics at 42°C for 24 h, and this was followed by a further 24 h of incubation at 50°C on LB plates without antibiotics. Finally, the positive colonies were transferred to LB plates with antibiotics to test for vector loss. The knockout mutants were those positive colonies that did not have vectors. Those positive colonies without the existence of vectors were considered to be the knockout mutants.

### Isolation and identification of secondary metabolites from *B. velezensis* DMW1.

The crude extract of secondary metabolites from DMW1 and its derivative mutants was obtained as described by Yu et al. and Wu et al. (54, 55). Subsequently, 5 μL of the crude extract was detected via high-performance liquid chromatography (HPLC, Waters), equipped with a reverse-phase column (ZORBAX SB-C18). The running program was a gradient elution from 5% solvent A (HPLC-grade acetonitrile containing 0.1% trifluoroacetic acid), 95% solvent B (Milli-Q water containing 0.1% trifluoroacetic acid) to 95% A, 5% B for 30 min. A concentration of 95% solvent A was then held for 10 min to elute all of the compounds. The detection of surfactin, fengycin, and iturin was done under UV absorption at 210 nm. While all of the polyketides (macrolactin, difficidin, and bacillaene) were detected under UV absorption at 280 nm, bacilysin was detected at 230 nm. For the purification of secondary metabolites, the semipreparative HPLC was used with a flow rate of 4 mL/min and an injection volume of 1 mL. The elution procedure was the same as the HPLC analysis. The DMW1 strain was used to isolate all non-ribosomal peptides (surfactin, fengycin, iturin, and bacilysin), whereas the triple-knockout mutant CJ5 was selected to isolate all three of the polyketides. At the last step, purified compounds were identified via ultraperformance liquid chromatography-mass spectrometry (UPLC-MS), using parameters as previously reported (54).

### Bioactivity assay.

The antagonistic activity against fungi and oomycetes of purified compounds produced by DMW1 was studied on PDA and 10% (vol/vol) V8 juice plates, respectively. A 6 mm diameter mycelial plug was placed in the center of a fresh PDA/V8 plate. Then, 7 mm diameter wells were made with a sterilized bore, 3 cm away from the center. Subsequently, 10 μL of the purified compound (50 μg/mL) were added to the wells and incubated at 25°C for 3 to 7 days. The percentage of inhibition was calculated with the following formula:

Inhibition rate (%)=[(C−T)×100)/C],

in which the control (C) and treatment (T) are the mycelial lengths (cm) of the fungus/oomycetes from the central mycelial plug to the side edge of the CK and each compound, respectively.

The *in vitro* bactericidal activities against the three bacteria were conducted in sterile 96-well microplates, using the broth microdilution method. Twofold serial dilutions of the target compound dissolved in methanol were prepared in sterile nutrient broth at concentrations ranging from 4 to 258 μg/mL. 100 μL of the dilution were mixed with 100 μL of bacterial suspension (1 × 10^6^ CFU/mL) and placed in the wells of a 96-well microplate. The microplates were incubated at 30°C for 24 h. Subsequently, 10 μL of 3-[4,5-dimethyl-2-thiazolyl]-2,5-diphenyl-2H-tetrazolium bromide (MTT) solution (100 μg/mL, dissolved in phosphate-buffered saline) were added to each well as an indicator. The lowest concentration at which no blue color was displayed was recorded as the MIC. Kanamycin sulfate in medium was used as a positive control, and methanol was used as a non-treated control. The experiment was repeated three times, and each experiment was performed with three replicates.

### Greenhouse experiments.

The soybean cultivar utilized in this experiment was HF47. The soybean seedlings were grown in the plant growth chamber at 25°C for 7 days in a 16 h daylight/8 h night cycle, after which 500 mL of LB medium or DMW1 cell suspension (10^6^ cells per mL) were drenched into each pot. 2 weeks later, to evaluate the promotion efficiency of the DMW1 and FZB42 strains, the stem height, root length, fresh weight, and dry weight of each plant were measured. Regarding the biocontrol effect of infection on soybean, the oomycete plant pathogen *P. sojae* 6497 was incubated on a 10% (vol/vol) V8 juice medium agar plate for 7 days, and the mycelium plugs of *P. sojae* were then mixed with sterilized soil. The soybean seedlings were soaked in LB medium or a bacterial suspension (10^6^ cells per mL) for 5 h and were subsequently sown in a pot that was filled with soil containing *P. sojae.* The survival rates of the soybean plants for each treatment were recorded under the aforementioned growth conditions for 2 weeks so as to determine the biocontrol effect of the DMW1 and FZB42 strains against *P. sojae*. The experiment was repeated two times and in three replicates, comprising 48 plants being studied under each treatment.

The tomato cultivar Moneymaker was sterilized, pre-bred, and grown as described in a previous report (56). In brief, the tomato seeds were surface sterilized in 75% ethanol, 20% sodium hypochloride and were then washed three times with sterile water. Subsequently, the tomato seeds were sown in nursery pots for 2 weeks and then transplanted into pots with 300 g of sterilized soil. After 2 weeks, 50 mL of LB medium or cell suspensions of *B. velezensis* (10^6^ cells per mL) were applied as irrigation to each pot. The growth conditions were a temperature of 28°C with a 16:8 h light/dark photoperiod. The stem length, stem thickness, fresh weight, and dry weight of plant aerial parts were measured 14 days after inoculation. As for the disease control experiment, 50 mL of an R. solanacearum cell suspension (10^6^ cells per mL) were drenched into each pot, 2 days after the DMW1 inoculation. After 14 days, the symptoms of tomato plants were photographed, and the disease index (DI) of each plant was recorded. The disease index was classified as follows: DI 0, no visible disease symptoms; DI 1, 1 to 30% of lesion areas; DI 2, 30 to 60% of lesion areas; DI 3, 60 to 90% of lesion areas; DI 4, >90% of lesion areas. Disease incidence was calculated according to the following formula:

Disease incidence=Σ(Ni×DIi)/(NT×hDI)×100.

The “Ni” term represents the number of diseased plants in disease index i (DIi, where i = 0, 1, 2, 3, 4). The “NT” and “hDI” terms represent the total number of investigated samples and the highest disease index, respectively.

The biocontrol efficacy was calculated according to the following formula: Biocontrol efficacy = (Disease incidence of control − Disease incidence of treated)/Disease incidence of control × 100%. The experiments above contained three replicates for each treatment, and each replicate consisted of 8 tomato plants. The experiment was repeated three times.

### Data availability.

The whole genomic sequence of *B. velezensis* DMW1 has been submitted to the GenBank database (http://www.ncbi.nlm.nih.gov/GenBank/) under accession number CP114180.
