# Supplementary material for: Profiling of Antimicrobial Metabolites Synthesized by the Endophytic and Genetically Amenable Biocontrol Strain Bacillus velezensis DMW1
Source: Microbiol Spectr. 2023 Feb 21;11(2):e00038-23. doi: 10.1128/spectrum.00038-23 (PMC10100683; doi:10.1128/spectrum.00038-23)
Supplement: Supplemental file 1 — Supplemental material. Download spectrum.00038-23-s0001.pdf, PDF file, 0.7 MB [file spectrum.00038-23-s0001.pdf]

Supplementary material

Profiling of antimicrobial metabolites synthesized by the endophytic and genetically amenable biocontrol strain, *Bacillus velezensis* DMW1

Chenjie Yu [a], Han Chen [a], Linli zhu [a], Yan Song [a], Qifan Jiang [a], Yaming Zhang [a], Qurban Ali [a], Qin Gu [a], Xuewen Gao [a], Rainer Borriss [b], Suomeng Dong[a], Huijun Wu [a]*

[a] Department of Plant Pathology, College of Plant Protection, Nanjing Agricultural University, Key Laboratory of Integrated Management of Crop Diseases and Pests, Ministry of Education, Nanjing, China

[b] Humboldt University Berlin, Institut für Biologie, 10115 Berlin; Germany

* Correspondence author

Chenjie Yu and Han Chen contributed equally to this work. Author order was determined both alphabetically and in order of increasing seniority.

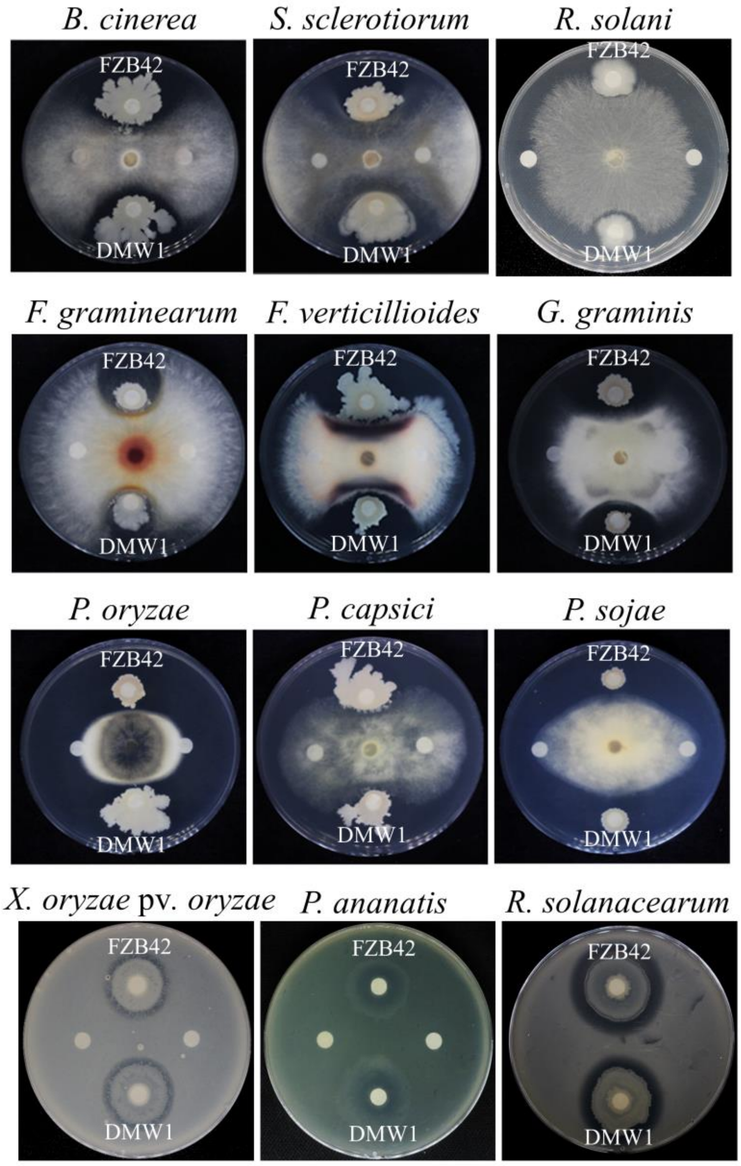

FIG S1 Inhibition effect of *B. velezensis* DMW1 and FZB42 against fungi (*B. cinerea*, *S. sclerotiorum*, *R. solani*, *F. graminearum*, *F. verticillioides*, *G. graminis*, *P. oryzae*), oomycetes (*P. capsica*, *P. sojae*), and bacteria (*X. oryzae* pv. *oryzae*, *P. ananatis*, *R. solanacearum*). The diameter of inhibition zone was recorded, and the inhibition rate was calculated.

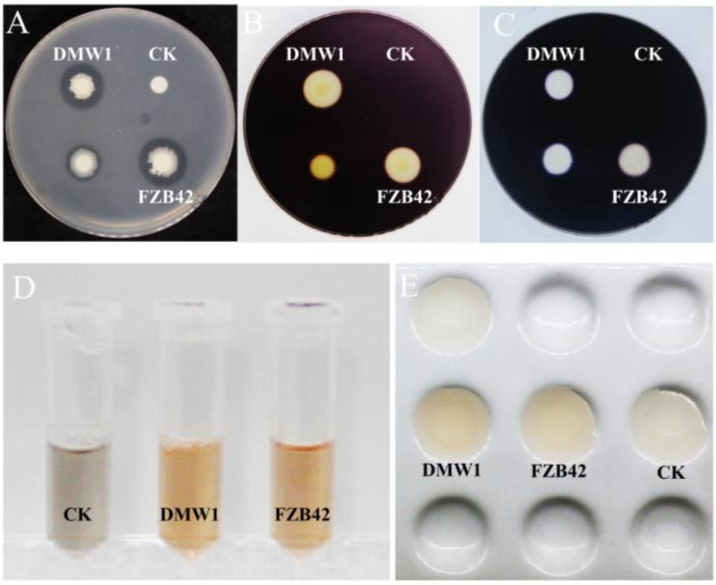

FIG S2 The detection of protease (A), cellulase (B) and amylase (C) activity, as well as siderophore (D) and IAA (E) production of *B. velezensis* DMW1 and FZB42. CK, liquid LB medium was set as the control. The clear halo zones represented the production of extracellular enzymes by DMW1 and FZB42. The changed color compared with CK represented the production of siderophore and IAA.

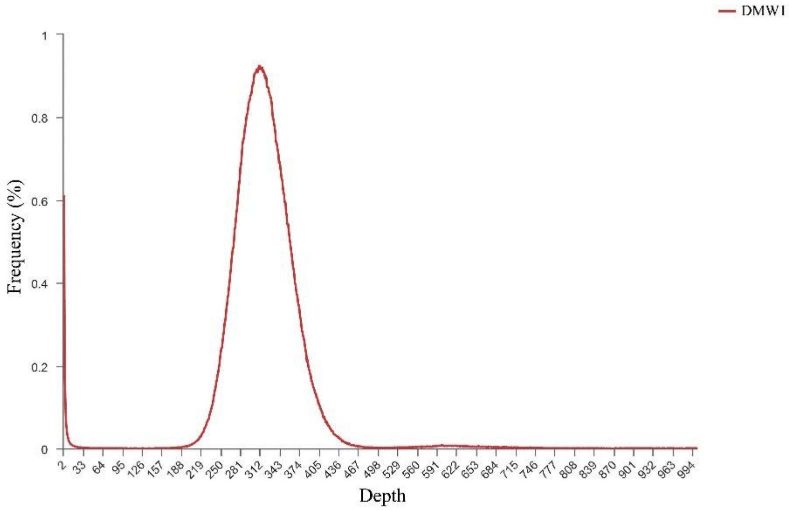

FIG S3 K-mer analysis of *B.velezensis* DMW1. X-coordinate represented depth and Y-coordinate represented proportion.

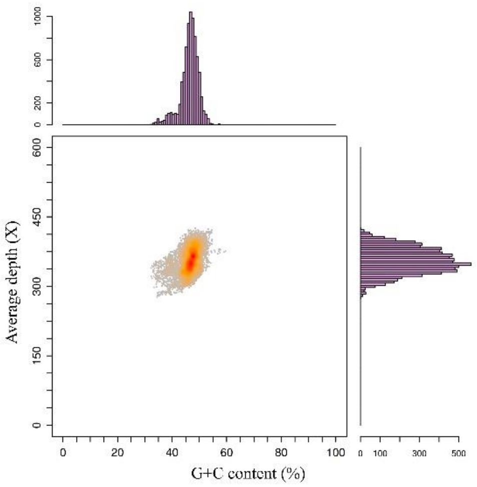

Fig S4 GC-depth analysis of *B.velezensis* DMW1. X-coordinate represented G+C content and Y-coordinate represented average depth.

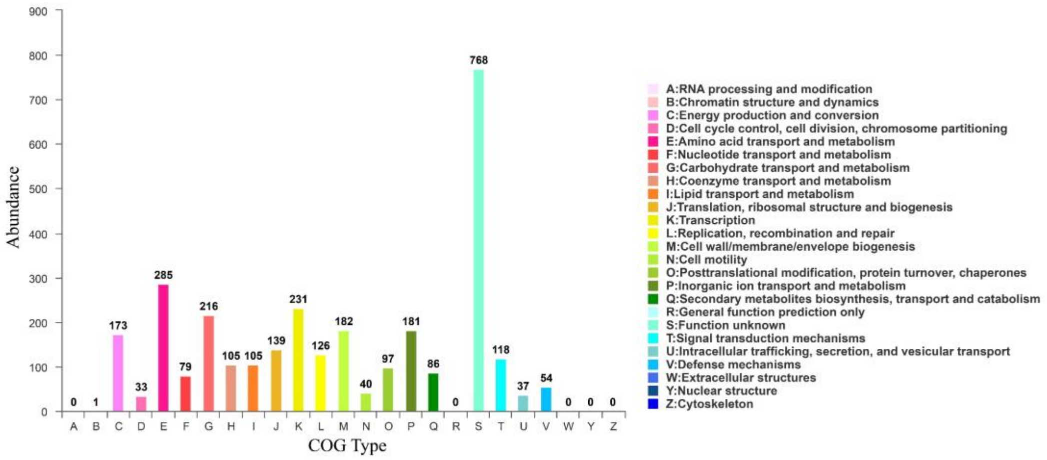

FIG S5 COG annotation of *B.velezensis* DMW1. Digital above each columns meant the number of genes divided to corresponding categories.

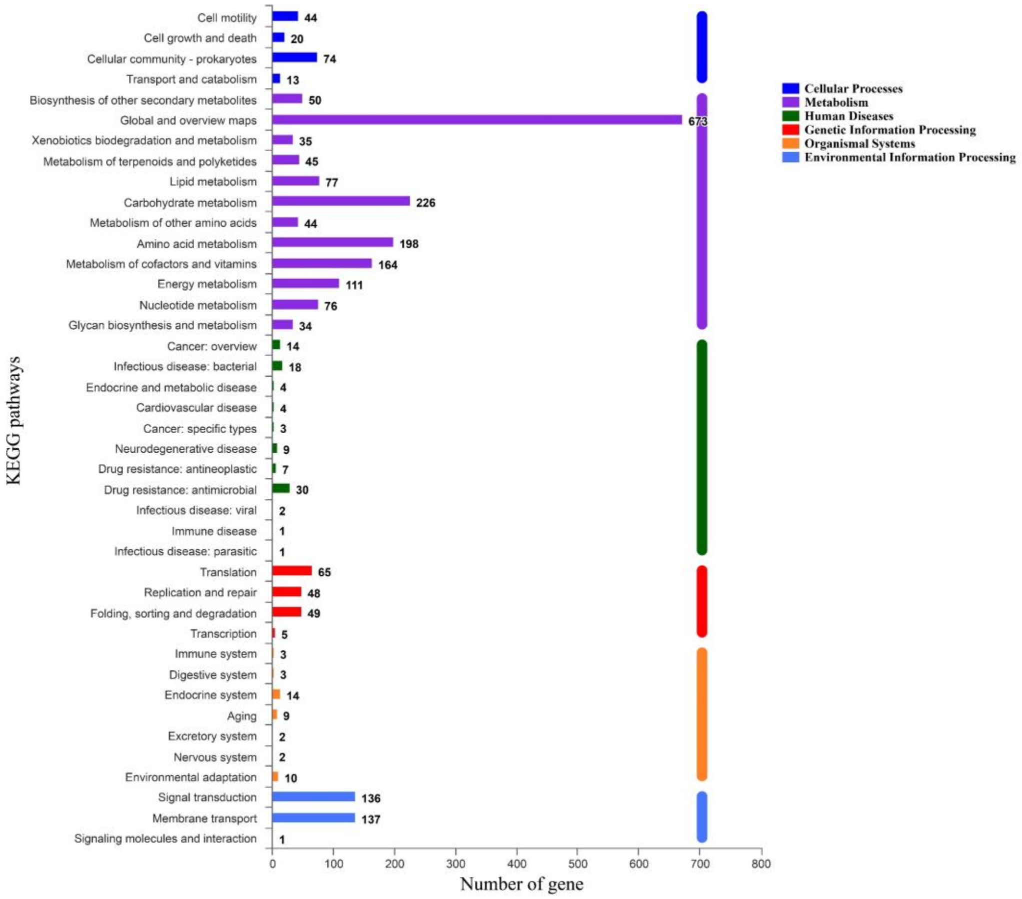

FIG S6 KEGG annotation of *B.velezensis* DMW1. Different colors represented the classification of the KEGG pathway.

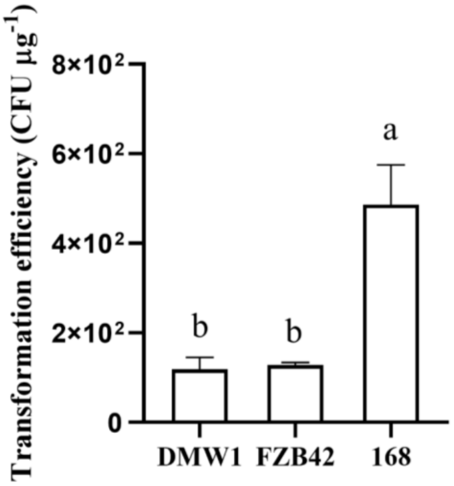

FIG S7 The detection of transformation efficiency of *B. velezensis* DMW1, *B. velezensis* FZB42 and *B. subtilis* 168. Data were analyzed by a one-way ANOVA, followed by Duncan's multiple range test. Different lowercase letter represented statistically significant differences (α=0.05)

TABLE S1 *In vitro* antagonistic activity of *B. velezensis* DMW1 and FZB42 against fungi, oomycetes and bacteria pathogens

Pathogen	Width of inhibition zone (cm)		Inhibition rate (%)	
	DMW1	FZB42	DMW1	FZB42
**Fungi**				
B. cinerea	2.90±0.06	2.90±0.10	70.15%	70.13%
S. sclerotiorum	2.63±0.09	2.50±0.06	64.73	61.46%
R. solani	2.63±0.03	2.37±0.07	64.22%	57.40%
F. graminearum	2.63±0.07	2.43±0.03	65.83%	61.16%
F. verticillioides	2.47±0.09	2.48±0.12	66.06%	65.88%
G. graminis	2.47±0.03	2.47±0.03	60.17%	60.17%
P. oryzae	1.07±0.07	1.07±0.07	30.72%	30.72%
**Oomycetes**				
P. capsici	2.23±0.09	2.17±0.03	63.18%	60.56%
P. sojae	1.70±0.06	1.73±0.15	48.09%	48.09%
**Bacteria**				
X. oryzae pv. *oryzae*	1.77±0.03	1.63±0.03	/	/
P. ananatis	1.00±0.00	0.87±0.03	/	/
R. solanacearum	2.30±0.12	2.50±0.06	/	/

TABLE S2 Genes associated with plant growth promotion in DMW1 genome

Gene	From	To	Product	Function
nasD	356,481	358,898	Nitrite reductase	Nitrogen fixation
nasE	356,140	356,460	Nitrite reductase small subunit	
narG	3,664,696	3,668,382	Nitrate reductase, alpha subunit	
narH	3,663,243	3,664,706	Nitrate reductase beta subunit	
nasA	363,654	364,859	Nitrate transporter	
narK	3,670,743	3,671,873		
dkgB	206,534	205,689	2,5-diketo-D-gluconic acid reductase	Phosphorus solubilization
speA	1,482,099	1,483,571	Arginine decarboxylase	Spermidine synthesis
speE	3,679,474	3,680,304	Spermidine synthase	
speH	2,810,449	2,810,835	S-adenosylmethionine decarboxylase	
bltD	600,115	600,573	Spermidine acetyltransferase	
ysnE	3,744,768	3,745,268	N-acetyltransferase	Indole-3-acetic acid synthesis
dhaS	2,052,357	2,053,844	aldehyde dehydrogenase	
yhcX	937,495	939,033	Nitrilase cyanide hydratase	
ilvH	2,732,268	2,732,786	Acetolactate synthase small subunit	3-hydroxy-2-butanone synthesis
ilvB	2,732,783	2,734,600	Acetolactate synthase large subunit	
alsD	3,551,453	3,552,220	Acetolactate decarboxylase	
alsS	3,552,281	3,553,996	Acetolactate synthase	
bdhA	653,815	654,855	Butanediol dehydrogenase	2, 3-butanediol synthesis
dhbF	3,081,520	3,088,647	Non-ribosomal peptide synthetase	Siderophore (bacillibactin) synthesis
dhbB	3,088,662	3,089,588	Isochorismatase	
dhbE	3,089,606	3,091,231	2,3-dihydroxybenzoate-AMP ligase	
dhbA	3,092,470	3,093,255	Dehydrogenase reductase	

TABLE S3 Strains and plasmids used in this study

Strains and plasmids	Relevant characteristics	Source
**Strains**		
DMW1	The wild type of *B. velezensis*	This study
FZB42	The wild type of *B. velezensis*	(1)
CJ1	DMW1 Δsrf, deficient in surfactin synthesis	This study
CJ3	DMW1 Δitu, deficient in iturin synthesis	This study
CJ12	DMW1 Δsrf fen, deficient in surfactin and fengycin synthesis	This study
CJ5	DMW1 Δsrf fen itu, deficient in surfactin, fengycin and iturin synthesis	This study
CJ6	DMW1 Δmln, deficient in marcolactin synthesis	This study
CJ7	DMW1 Δdfn, deficient in difficidin synthesis	This study
CJ8	DMW1 Δbae, deficient in bacillaene synthesis	This study
CJ9	DMW1 Δbac, deficient in bacilysin synthesis	This study
B. cinerea		This lab
S. sclerotiorum		This lab
R. solani		This lab
F. graminearum PH-1	Phytopathogenic fungi	This lab
F. verticillioides 7600		This lab
G. graminis		This lab
P. oryzae		This lab
P. capsica	Phytopathogenic oomycetes	This lab
P. sojae P6497		(2)
X. oryzae pv. *oryzae* PXO99^{A}	Phytopathogenic bacteria	(3)
P. ananatis DZ-12		This lab
R. solanacearum		This lab
**plasmids**		
pJOE8999	Wild type vector for CRISPR/Cas9 genome editing	(4)
pAD 43-25	GFP-plasmid for the detection of transformation efficiency.	This lab
pJOEΔsrf	Knock-out vector for gene of surfactin synthesis	This study
pJOEΔitu	Knock-out vector for gene of iturin synthesis	This study
pJOEΔfen	Knock-out vector for gene of fengycin synthesis	This study
pJOEΔmln	Knock-out vector for gene of marcolactin synthesis	This study
pJOEΔdfn	Knock-out vector for gene of difficidin synthesis	This study
pJOEΔbae	Knock-out vector for gene of bacillaene synthesis	This study
pJOEΔbac	Knock-out vector for gene of bacilysin synthesis	This study

TABLE S4 Primers used in this study

Primer name	Sequence (5' to 3')	Purpose
sg-srf-F	tacgagaaacgctttaccgcacag	Construction of pJOE⊿srf
sg-srf-R	aaacctgtgcggtaaagcgtttct	
srfL-F	cactatagggtcgacggccaacgaggcctgatatggaggacgtccatgttt	
srfL-R	taatgcgcgagccatgtagtctacatgcttgacc	
srfR-F	actacatggctcgcgcattaaaaaagagctc	
srfR-R	ttcttaatctagaaaggccttattggcccgggcttcctccttttctatcg	
sg-fen-F	tacgtcgggaactgaaacaaatcg	Construction of pJOE⊿fen
sg-fen-R	aaaccgatttgtttcagttcccga	
fenL-F	cactatagggtcgacggccaacgaggccttgatcatgaagatcaatacgatattt	
fenL-R	tgcagatcttagcaagcctgatctccggtt	
fenR-F	caggcttgctaagatctgcagacagcttatcagc	
fenR-R	ttcttaatctagaaaggccttattggcctgaagaactcgattgccccc	
sg-itu-F	tacggactccgtctcattacccgg	Construction of pJOE⊿itu
sg-itu-R	aaacccgggtaatgagacggagtc	
ituL-F	cactatagggtcgacggccaacgaggccggcatcaaatgttttacggcc	
ituL-R	agggtattggagatcgatcccgaacggttaaa	
ituR-F	ggatcgatctccaataccctctggcaacacc	
ituR-R	ttcttaatctagaaaggccttattggccgtcggaattttggctgttctga	
sg-bae-F	tacggccgtgaatgaaacagacgg	Construction of pJOE⊿bae
sg-bae-R	aaacccgtctgtttcattcacggc	
baeL-F	cactatagggtcgacggccaacgaggcctgggacttatcttccatgcgtt	
baeL-R	atgcggcaattctctgcagaagccgtgtct	
baeR-F	tctgcagagaattgccgcatcccgttcga	
baeR-R	ttcttaatctagaaaggccttattggccatgccgatgagatcatcaggg	
sg-mln-F	tacgggacagaaaagattaatcag	Construction of pJOE⊿mln
sg-mln-R	aaacctgattaatcttttctgtcc	
mlnL-F	cactatagggtcgacggccaacgaggcccagctttattgaagcccacgg	
mlnL-R	atatatcccgttgcctcctgatacccaaaatt	
mlnR-F	caggaggcaacgggatatatcagaaccgctga	
mlnR-R	ttcttaatctagaaaggccttattggccgcttcctcagaaagctctgacc	
sg-dfn-F	tacgggagatatacggaaaaacag	Construction of pJOE⊿dfn
sg-dfn-R	aaacctgtttttccgtatatctcc	
dfnL-F	cactatagggtcgacggccaacgaggcccagtgatcgcgaccaatgtg	
dfnL-R	ctttcagcttgctcggaatcttcagcgtttt	
dfnR-F	gattccgagcaagctgaaaggatggatccgc	
dfnR-R	ttcttaatctagaaaggccttattggccgaaggggcaatcctcttatgttt	
sg-bac-F	tacgcgaagcgttcaacagcattg	Construction of pJOE⊿bac
sg-bac-R	aaaccaatgctgttgaacgcttcg	
bacL-F	cactatagggtcgacggccaacgaggcccagtgatcgcgaccaatgtg	
bacL-R	ctttcagcttgctcggaatcttcagcgtttt	
bacR-F	gattccgagcaagctgaaaggatggatccgc	
bacR-R	ttcttaatctagaaaggccttattggccgaaggggcaatcctcttatgttt	

KOsrf-F	atttaacggcttgctcaagc	
KOsrf-R	gctgttcaaaatcctcaacg	
KOfen-F	aaaccgttcgattgaagcga	
KOfen-R	atccagaagcgaatgaaaca	
KOitu-F	gaataaaggattgcggcttg	
KOitu-R	gttacggacaaaaggcgtac	Verification of the knock out of each gene
KObae-F	accgagcgaatccattttga	
KObae-R	gaaatcctgctgtcggcgat	
KOmln-F	gtgtacgcagtcattaaagg	
KOmln-R	aattgagatcctcttcacgc	
KOdfn-F	tgaagcgcatattgccgaat	
KOdfn-R	gtcgtttccattccgtttct	
KObac-F	cggcacagcttatttctgcg	
KObac-R	aatccggccctgaatctggt	

Reference

1. Koumoutsi A, Chen XH, Henne A, Liesegang H, Hitzeroth G, Franke P, Vater J, Borriss R. 2004. Structural and functional characterization of gene clusters directing nonribosomal synthesis of bioactive cyclic lipopeptides in *Bacillus amyloliquefaciens* strain FZB42. Journal of Bacteriology 186:1084-1096.
2. Connolly MS, Williams N, Heckman CA, Morris PF. 1999. Soybean isoflavones trigger a calcium influx in *Phytophthora sojae*. Fungal Genetics and Biology 28:6-11.
3. Hopkins CM, White FF, Choi SH, Guo A, Leach JE. 1992. Identification of a family of avirulence genes from *Xanthomonas-oryzae* pv *oryzae*. Molecular Plant-Microbe Interactions 5:451-459.
4. Altenbuchner J. 2016. Editing of the *Bacillus subtilis* genome by the CRISPR-Cas9 system. Applied and Environmental Microbiology 82:5421-5427.
